# Diagnostic Techniques to Increase the Safety of Phakic Intraocular Lenses

**DOI:** 10.3390/diagnostics13152503

**Published:** 2023-07-27

**Authors:** Tadas Naujokaitis, Gerd U. Auffarth, Grzegorz Łabuz, Lucy Joanne Kessler, Ramin Khoramnia

**Affiliations:** Department of Ophthalmology, University of Heidelberg, 69120 Heidelberg, Germany

**Keywords:** anterior chamber depth, cataract, endothelial cell density, endothelial cell loss, iris-fixated pIOL, glaucoma, phakic intraocular lens, pIOL, posterior chamber, vault

## Abstract

Preoperative and postoperative diagnostics play an important role in ensuring the safety of patients with phakic intraocular lenses (pIOLs). The risk of endothelial cell loss can be addressed by regularly measuring the endothelial cell density using specular microscopy and considering the endothelial cell loss rate and the endothelial reserve in accordance with the patient’s age when deciding whether to explant a pIOL. The anterior chamber morphometrics, including the anterior chamber depth and the distance between the pIOL and the endothelium, measured using Scheimpflug tomography and anterior segment optical coherence tomography (AS-OCT), can help to assess the risk of the endothelial cell loss. In patients undergoing posterior chamber pIOL implantation, accurate prediction of the vault and its postoperative measurements using AS-OCT or Scheimpflug tomography are important when assessing the risk of anterior subcapsular cataract and secondary glaucoma. Novel approaches based on ultrasound biomicroscopy and AS-OCT have been proposed to increase the vault prediction accuracy and to identify eyes in which prediction errors are more likely. Careful patient selection and regular postoperative follow-up visits can reduce the complication risk and enable early intervention if a complication occurs.

## 1. Introduction

Throughout the past seven decades of phakic intraocular lens (pIOL) development, one of the main areas of focus has been reducing the risk of postoperative complications [1]. This led to the creation of numerous different pIOL designs [1]. However, most of these later disappeared from the market because of safety concerns [1,2]. The few pIOL models currently available are based on designs that were proven successful over a development and observation period of more than two decades: iris-fixated anterior chamber pIOLs and posterior chamber pIOLs [2].

A variety of postoperative complications can occur in patients with pIOLs, including pupil ovalization, uveitis, hyphema, pIOL dislocation, synechiae formation, dysphotopsia, pupillary block glaucoma, pigment dispersion, anterior subcapsular cataract formation, and endothelial damage [1,2,3,4,5,6,7]. While the complication rate has been reduced by developing new materials, optimizing pIOL designs, and improving manufacturing techniques, there is an increasing awareness that patient selection and postoperative monitoring also play an important role in ensuring the safety of patients with pIOLs [1,4,5,7,8,9,10].

The aim of this review is to discuss how the preoperative and postoperative diagnostic techniques can be utilized to reduce the complication risk in patients with pIOLs, in particular that of endothelial cell loss, cataract development, and glaucoma, which are among the main concerns with the currently available pIOL models [6].

## 2. Types of Phakic Intraocular Lenses

Knowledge of different pIOL designs is important in order to understand how to address the potential complications. In general, pIOLs can be classified as those implanted into the anterior chamber and into the posterior chamber [2]. The former can be either iris-fixated or angle-supported [1,2].

### 2.1. Iris-Supported Anterior Chamber Phakic Intraocular Lenses

There are two iris-fixated pIOL models available on the market: Artisan (Ophtec B.V., Groningen, the Netherlands), which is also known as Verisyse (Johnson & Johnson Surgical Vision, Inc., Santa Ana, CA, USA); and Artiflex (Ophtec B.V.), also known as Veriflex (Johnson & Johnson Surgical Vision, Inc.) [10]. The Artisan pIOL is a non-foldable lens with an optic diameter of 5.0 to 6.0 mm, manufactured from polymethyl methacrylate (PMMA) and used to correct myopia, hyperopia, and astigmatism [2,10,11]. The Artiflex pIOL was developed to reduce the incision size needed for the implantation of the lens and features a flexible 6.0 mm silicone optic with rigid PMMA haptics [2,10]. It requires a corneal incision of 3.1 mm, which is considerably smaller than the 5.2–6.2 mm incision needed to implant the Artisan lens [2]. However, it is only available for the correction of myopia and astigmatism [2,12]. The Artisan lens was made available more than 30 years ago, while the Artiflex lens was introduced onto the market a decade later [10,13,14]. Therefore, long-term safety data are available for both iris-fixated pIOL models [10,12,14,15,16,17,18,19,20,21,22,23,24]. Although the rate of reported postoperative complications is low, there are concerns regarding endothelial cell loss, which is considerable in some patients [5,19].

### 2.2. Angle-Supported Anterior Chamber Phakic Intraocular Lenses

Due to their ease of implantation, many of the early intraocular lens (IOL) models were anterior chamber angle-supported lenses [1]. The developed phakic angle-supported IOL designs included rigid PMMA lenses, partially foldable lenses featuring a combination of silicone optic and PMMA haptics, and foldable hydrophilic and hydrophobic acrylic lenses [1,2,7,10,25,26]. However, many of them were associated with an unacceptable rate of complications, which included chronic uveitis, pupillary ovalization, synechiae, and endothelial damage. This led to these lenses being taken off the market [2,10,27]. One of the most popular angle-supported pIOL models, although it is no longer implanted, was the hydrophobic acrylic Acrysof Cachet (Alcon Laboratories, Inc, Fort Worth, TX, USA) pIOL for the correction of myopia [2,27]. In contrast to the iris-fixated lenses, the Acrysof Cachet was designed to be able to rotate in the anterior chamber in order to minimize the compression-induced damage to the anterior chamber structures, and a non-toric version of the lens was available for this reason [27]. The single-piece design featured a 6.0 mm optic with an overall lens length of 12.5 to 14.0 mm [2]. Due to safety concerns regarding endothelial cell loss, it was voluntarily withdrawn from the market by its manufacturer [27]. However, it is still common to see patients implanted with the Acrysof Cachet pIOL in clinical practice.

### 2.3. Posterior Chamber Phakic Intraocular Lenses

Posterior chamber pIOLs are implanted between the iris and the crystalline lens, with the haptics positioned in the ciliary sulcus [2,28]. Most data available regarding the safety of posterior chamber pIOLs are that of the Implantable Collamer Lens (ICL; STAAR Surgical AG, Nidau, Switzerland). It is a foldable lens with plate haptics, manufactured using a proprietary Collamer material, and it is used to correct myopia, hyperopia, and astigmatism [29,30,31]. Early studies reported considerable rates of anterior subcapsular cataract in patients implanted with ICLs [32]. These safety concerns were addressed by several design changes, most notably the introduction of the central port with a 0.36 mm diameter in the V4c model [31]. The lens is available in four sizes, with overall lengths of 12.1, 12.6, 13.2, and 13.7 mm for the V4c model [33].

In recent years, new posterior chamber pIOL models have been introduced, but their long-term outcomes are yet to be evaluated [34,35]. The Eyecryl (Biotech Vision Care Pvt Ltd., India) pIOL is made using hydrophilic acrylic material and features a very similar design to the ICL; the size of its central port is identical to the one found in the V4c model [35]. The Implantable Phakic Contact Lens (IPCL; Care Group, Baroda, India) is made using reinforced hybrid acrylic material with medium water content, and its current version features two holes in the superior part of the optic, as well as a central hole in lenses with negative refractive powers and in plus power lenses of up to +3.5 D [34,36]. In addition to its ability to correct myopia, hyperopia, and astigmatism, a multifocal diffractive presbyopia-correcting version of this pIOL is available [36,37].

## 3. Addressing the Risk of Endothelial Cell Loss

The main issue with the first anterior chamber IOL designs was their high rate of corneal decompensation [1]. Despite decades of pIOL development, the endothelial damage caused by the lenses has remained a significant issue even in some modern pIOLs, such as the angle-supported Acrysof Cachet pIOL [5,10]. Kohnen et al. reported a mean yearly rate of chronic endothelial cell loss of 1.7% in patients implanted with this pIOL, and 10.3% of these patients’ eyes underwent the explantation of the lens, mostly because of endothelial cell loss [38].

The underlying cause for the endothelial cell loss observed in patients with pIOLs is probably related to mechanical damage to the corneal endothelium. This is apparent when analyzing the cases of dislocated iris-fixated pIOLs [24,39]. Mechanical damage to the endothelium due to direct contact with the pIOL can also occur when rubbing the eyes and may, in some cases, even require the explantation of the lens [20]. Nevertheless, high rates of endothelial cell loss are also sometimes observed in eyes without a history of trauma, pIOL dislocation, or eye rubbing, suggesting that there are other factors influencing endothelial cell loss in patients with pIOLs [20,24].

While the conventional slip lamp examination is an essential part of the ophthalmological examination and helps to detect pathologies such as pIOL subluxation or corneal edema due to endothelial decompensation, additional diagnostic modalities are necessary in order to obtain a complete picture of the factors influencing endothelial cell loss in pIOL patients.

### 3.1. Endothelial Cell Density Measurement

As significant endothelial cell losses are occasionally observed in patients with pIOLs, it is essential to regularly monitor the endothelium of each patient [9,10,40]. In a healthy and young cornea, the endothelium consists of a single layer of finite hexagonal cells of a similar size [41,42]. Its main function is controlling the hydration and nutrition of the cornea, which it achieves by allowing some aqueous humor and nutrients to pass through into the stroma and by actively pumping excess water out of the stroma to maintain a clear cornea [41,42]. Due to the limited regenerative capacity of human corneal endothelial cells, their number continuously decreases with age, and the coverage of the posterior corneal surface is ensured by cellular migration from the periphery, where the cell density is higher, into the central cornea, while the cell area enlarges to avoid gaps between cells [41,43,44]. When endothelial damage occurs, such as that occasionally observed in patients with pIOLs, the loss of endothelial cells is compensated by the enlargement of the remaining cells and other morphological changes, such as the loss of the hexagonal shape of some cells [45]. In cases where the endothelial damage is severe and the function of the remaining cells is insufficient, corneal endothelial decompensation occurs, leading to corneal edema [44].

**Figure 1:**
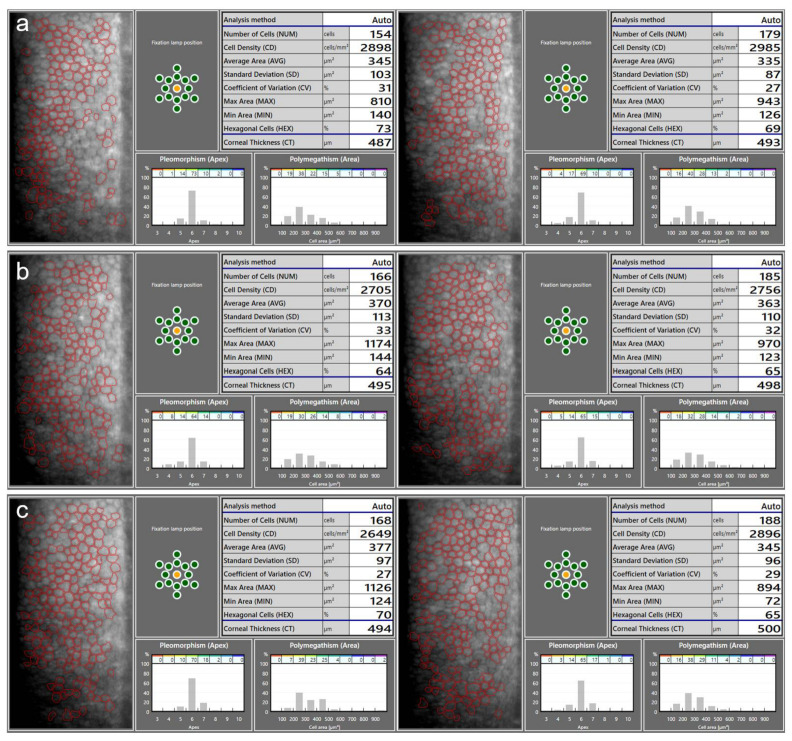
An example of ECD measurements using a specular microscope CEM-530 (Nidek, Gamagori, Japan) on a patient before the implantation of the anterior chamber iris-fixated phakic intraocular lenses (pIOLs) in both eyes (**a**), as well as 3 months (**b**) and 1 year (**c**) following the surgeries. At 1 year post operation, the slight decrease in the measured ECD in the OD and the increase in the OS are within the range of the expected variability in the ECD measurement. Repeated measurements over several follow-up visits are necessary to determine the actual changes in the ECD. Images on the left: OD; images on the right: OS.

The most common technique for assessing the corneal endothelium of patients with pIOLs is specular microscopy (Figure 1). It provides a high-magnification view of the specular light reflected from the endothelium and allows for the examination of the morphology of the corneal endothelial cells [45,46]. The modern specular microscopes can perform non-contact automated measurements and evaluate different parameters, such as the endothelial cell density (ECD), mean cell area, coefficient of variation, and percentage of hexagonal cells [41,46]. The most commonly used metric is the central ECD, expressed as the number of cells per square millimeter [45].

**Figure 2:**
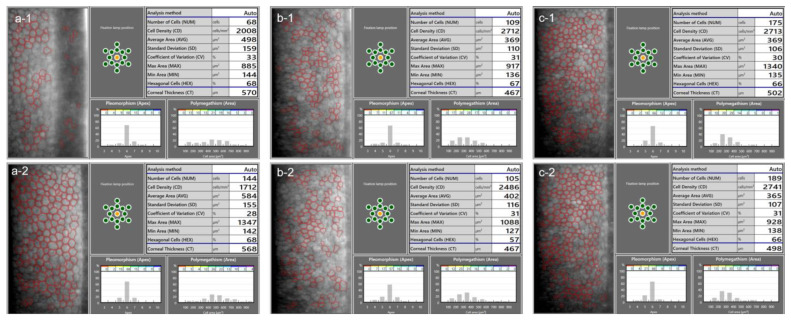
In a low-quality specular microscopy image (**a-1**), the ECD was overestimated by the automatic algorithm. The primary reason for this is that the artefacts were recognized as small endothelial cells by the algorithm, decreasing the average value of the cell area. The ECD is based on the average measured cell area, and the underestimation of this value resulted in an overestimated ECD. In a repeated measurement (**a-2**), a good quality image was obtained, and the algorithm could correctly recognize most of the cells. The repeatability of ECD measurement is generally low when few cells are recognized, which is apparent from an inspection of the specular microscopy images from another patient (**b-1**,**b-2**). In cases where the images are of good quality, however, the automated measurement can provide good repeatability (**c-1**,**c-2**, another patient).

**Figure 3:**
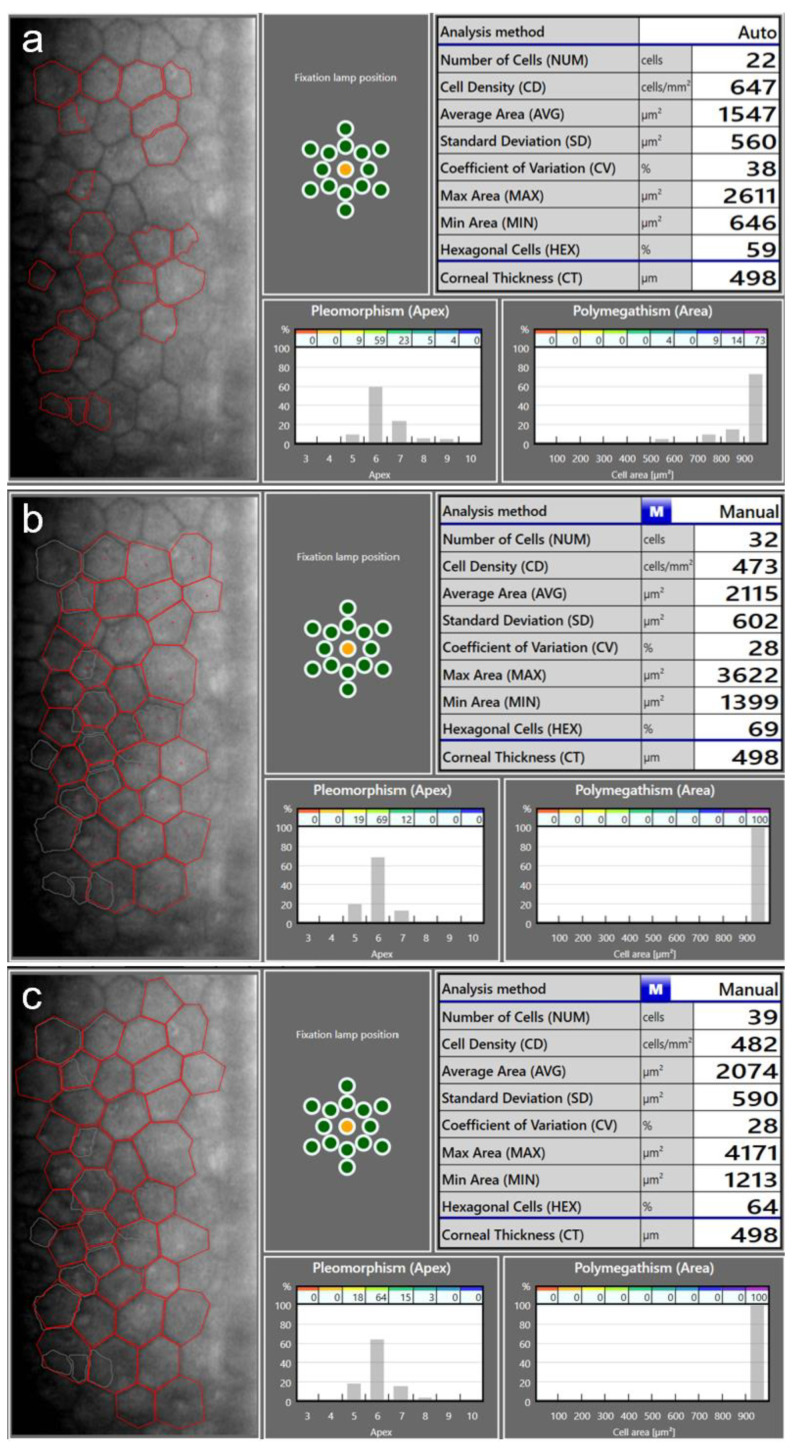
Endothelial cell density (ECD) measurement in a patient with a very low ECD. An automatic measurement (**a**) overestimated the ECD (674 cells/mm^2^) as the algorithm did not recognize the cell boundaries correctly. Using the same image, manual measurements using the center method (**b**) and the corner method (**c**) resulted in lower ECD values (473 cells/mm^2^ and 482 cells/mm^2^, respectively).

There are several aspects to be aware of when measuring ECD using specular microscopy. In order to obtain a reliable ECD measurement, a quality image of the endothelium is necessary, ideally while maintaining well-focused and clearly identifiable cells throughout the whole image. Low-quality images may lead to incorrect recognition of cell boundaries, especially by the automated algorithms (Figure 2). As the ECD value depends on the average measured or estimated cell area, a false identification of several cells as one would lead to the underestimation of the ECD, and vice versa [45]. A study by Huang et al. found that automated ECD analysis using the Konan specular microscope NSP-9900 (Konan Medical USA Inc., Irvine, CA, USA) significantly overestimated ECD in eyes with a large cell size (i.e., low ECD) and in those with a high polymegathism (i.e., cell size variation) [47]. In one of their examples, the fully automated algorithm provided an ECD value of 2410 cells/mm^2^, while the ECD value based on manual analysis was only 728 cells/mm^2^ [47]. It is therefore essential to visually inspect each specular microscopy image to assess the quality and check for errors in automated cell recognition. If necessary, a manual analysis can be performed (Figure 3). An unreliable measurement can also be a result of a low number of cells being recognized [45]. The maximum number of cells which can be captured per image depends on both the field of view on the actual specular microscope used and the ECD [45]. In general, it is advisable to capture three images from the area analyzed, with as many cells visible in a single image as possible [45]. The repeatability of the ECD measurement is an important issue to consider when determining a change in ECD [10]. Studies have reported a variability in a repeatedly measured ECD of over 200 cells/mm^2^, and a standard deviation between the repeated measurements of up to 84 cells/mm^2^ [10,48,49]. In order to acquire more accurate ECD values, repeated ECD measurements can be averaged. Furthermore, the use of different specular microscopes can introduce additional errors, as systematic differences exist between ECD measurements obtained using different devices [49,50,51].

In addition to specular microscopy, the ECD can be measured using confocal microscopy [52]. However, it is not routinely performed in pIOL patients as it is a contact measurement and is more time-consuming than automated specular microscopy. A study by Fliotsos et al. demonstrated that it is also possible to measure the ECD using a slit lamp and a smartphone, although further research is needed to evaluate the reliability of smartphone-based ECD analysis [53].

### 3.2. Assessing Endothelial Cell Loss

The average central ECD in an adult is around 2500–3000 cells/mm^2^, and it physiologically decreases with age at the rate of 0.5–0.6% per year [44,47,54,55]. Normally, endothelial cell loss is not noticeable. In patients with pIOLs, the average yearly endothelial cell loss rate is usually up to 1% or 2%, but it can also be considerably higher [10,56]. Endothelial decompensation with corneal edema can occur if a critical ECD of about 400–700 cells/mm^2^ is reached [44,57].

The concept of the endothelial reserve is essential to understanding the implications of endothelial cell loss in pIOL patients. In practice, one may consider it as the difference between the current ECD in a particular patient and the critical ECD of 400–700 cells/mm^2^ [10,44]. To ensure the safety of patients with pIOLs, it is important to avoid reaching this critical ECD at any point throughout their lives [10]. In addition to physiological endothelial cell loss and the additional chronic loss which can be caused by a pIOL, acute losses due to the surgical trauma need to be considered as well [10]. While the pIOL implantation itself can cause endothelial cell loss, it is usually limited to 4–8% [11,16,18,22,28,58,59,60,61,62,63,64]. Larger losses can occur during pIOL explantation, alone or when combined with the cataract surgery [21,65,66,67]. One study found a statistically significant endothelial cell loss (27% decrease in mean ECD) in patients with iris-fixated lenses when cataract was the indication for the combined surgery, but not in those who underwent the combined surgery because of endothelial cell loss, suggesting that endothelial damage due to phacoemulsification is the primary reason for the decreased ECD [10,67]. However, another study reported a high average loss of more than 25% after the explantation of iris-fixated pIOLs without cataract surgery [21]. No statistically significant endothelial cell loss was observed after the explantation of posterior chamber pIOLs in combination with cataract surgery [68]. These discrepancies among studies could result from differences in surgical techniques as well as individual factors such as the type of cataract and the hardness of the lens, indicating the need for further research in this area [10].

The concept of the endothelial reserve should be considered when deciding whether a pIOL is safe to implant in a particular patient [10]. Younger patients will experience a larger cumulative endothelial cell loss than older patients, and therefore, a higher preoperative ECD is required in younger patients [10,59]. Bouheraoua et al. developed a model for the average ECD decrease after an iris-fixated pIOL implantation and found that the minimum preoperative ECD necessary for a 20-year-old patient is >3620 cells/mm^2^, which would ensure a density of at least 1500 cells/mm^2^ at the age of 70 years. Meanwhile, the ECD required for a 35-year-old was >2800 cells/mm^2^ [59]. An ECD value of 1500 cells/mm^2^ is mentioned by some authors as the minimum acceptable ECD in patients with pIOLs and the point at which the cataract surgery can still be safely performed [20,59,66,69]. However, a higher ECD before pIOL explantation or cataract surgery may be desirable, as a larger endothelial reserve could help to avoid the critical ECD being reached, even in cases of high endothelial cell loss [10]. When determining the point at which the ECD is unacceptable and the pIOL should be explanted, one should also consider the further physiological endothelial cell loss after the lens explantation and ensure that there is a sufficient endothelial cell reserve in case further intraocular interventions are needed later in life [10]. For example, Kim et al. reported that mean (±SD) ECD decreased from 1375 ± 468 cells/mm^2^ to 1020 ± 369 cells/mm^2^ after the explantation of iris-fixated pIOLs—an ECD value which is sufficient to avoid immediate postoperative endothelial decompensation, but which could increase the risk of corneal decompensation later in life, such as after cataract surgery.

Therefore, instead of delaying pIOL explantation until the ECD decreases to 1500 cells/mm^2^, it seems more appropriate to act as soon as an unacceptable endothelial cell loss rate is confirmed [10,40]. Due to the limited repeatability of ECD measurement, a reasonable approach is to shorten the follow-up intervals in case of a higher-than-expected endothelial cell loss and explant the lens if the tendency toward the ECD decreasing at an unacceptable rate continues [10,40]. When determining what rate is unacceptable in the particular patient, one should consider the age of the patient, as well as taking into account the expected ECD loss that may occur during pIOL explantation and cataract extraction performed later in the patient’s life [10]. A younger person would require a higher endothelial reserve because of a longer life expectancy, and therefore, the maximum tolerated endothelial cell loss rate may be lower [10].

### 3.3. Endothelial Cell Loss and the Phakic Intraocular Lens Type

In general, the endothelial cell losses in most patients with ICLs are low [15,58,61,62,70,71,72,73,74,75,76,77]. Several studies reported either no endothelial cell loss or low loss rates in the first few years following the ICL implantation, with stable ECD values afterwards, indicating that the decrease in ECD may be primarily related to endothelial remodeling following the surgery and not chronic endothelial damage inflicted by the lens itself [10,15,58,70,71,72,78]. Two studies reported high mean rates of endothelial cell loss: 21.8% after 5 years and 21.7% after 10 years [61,76]. The authors do not explain the reason for the unusually high losses, but the influence of the low sample size (14 eyes with long-term data) used in one of these studies should be considered [61,76]. In general, severe endothelial damage seems to be rare in patients with posterior chamber pIOLs; no lenses were explanted due to endothelial cell loss in the analyzed long-term studies [15,58,61,62,70,71,72,73,74,75,76,77]. In contrast, it is sometimes necessary to explant anterior chamber iris-fixated pIOLs because of endothelial cell loss, with reported explantation rates of up to 3.2% in most long-term studies with a postoperative follow-up period of up to 10 years, and a very high rate of 26% in one study with a shallow mean anterior chamber depth (ACD) [11,14,16,18,19,20,22,28,59,60,63,79,80,81,82,83,84]. Despite the lack of high-quality comparative long-term studies of endothelial cell loss in patients with anterior chamber pIOLs vs. posterior chamber pIOLs, the currently available data indicate a tendency towards lower losses in patients with posterior chamber pIOLs, which is probably due to a larger distance between the pIOL and the corneal endothelium in comparison to that with the anterior chamber lenses [10]. A summary of studies presenting long-term data on endothelial cell density changes in patients with pIOLs is presented in Table 1.

### 3.4. Anterior Chamber Morphometrics

**Figure 4:**
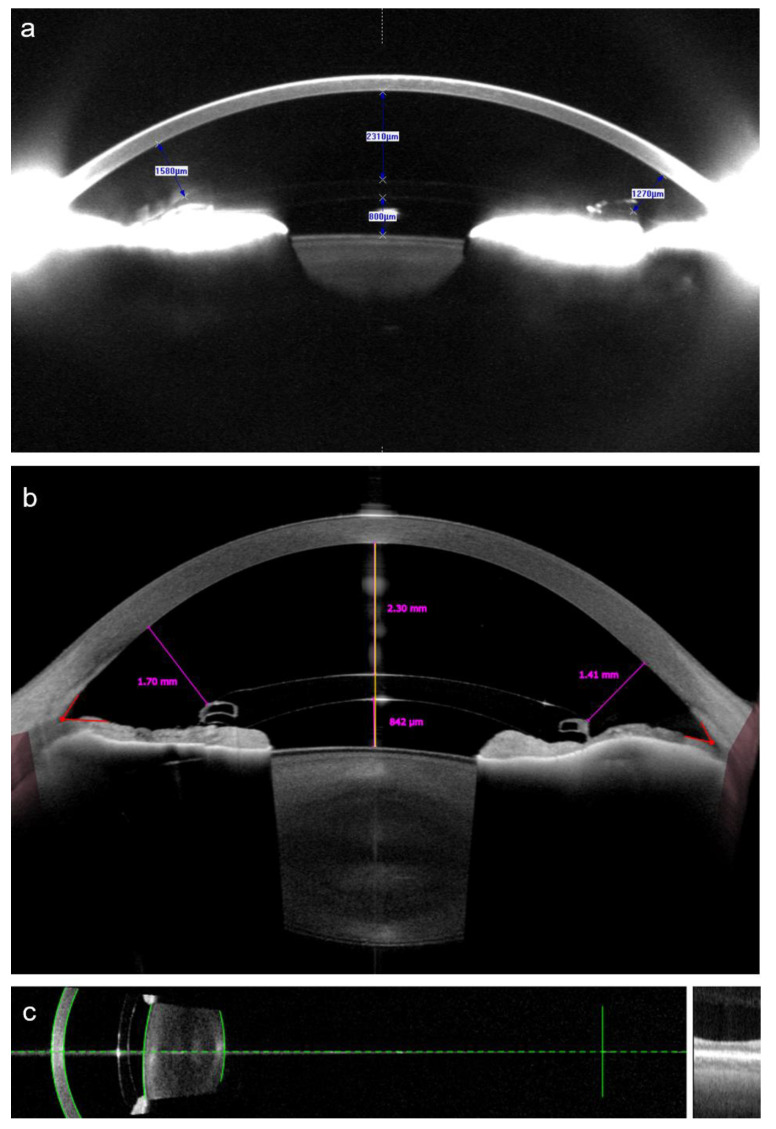
Analysis of the anterior chamber morphometrics in a patient with an anterior chamber iris-fixated pIOL using Scheimpflug tomography (**a**), obtained using Pentacam (Oculus Optikgeräte, Wetzlar, Germany); and the swept-source anterior segment optical coherence tomography (**b**), obtained using Anterion (Heidelberg Engineering, Heidelberg, Germany). Both techniques provided nearly identical measurements of the central distance between the pIOL and the corneal endothelium, while the measured distances between the pIOL edge and the endothelium were smaller when using Scheimpflug tomography. The same patient was examined using an optical biometer IOL Master 700 (Carl Zeiss Meditec, Jena, Germany) (**c**), also based on the swept-source optical coherence tomography technology. The device displays the automatically recognized surfaces as green lines, allowing the examiner to check whether the ocular structures were identified correctly.

Anterior segment imaging techniques can be used to assess the relationship between a pIOL and the ocular structures (Figure 4). One of the modalities available for this purpose is the ultrasound biomicroscopy (UBM) [85]. Due to its high frequency of approximately 40 to 50 MHz, it achieves a fivefold higher precision in comparison to B-scan ultrasound measurements of the posterior segment and can be used to determine the position of pIOLs [85,86,87]. UBM was introduced in the 20th century and is still unmatched in its ability to visualize structures behind the iris, such as the ciliary body [87]. However, it is now rarely used for anterior chamber analysis as newer technologies enable non-contact measurements. One of the most common techniques for this purpose is Scheimpflug photography, also called Scheimpflug tomography. It allows for the assessment of the position of angle-supported, iris-fixated, and posterior chamber pIOLs and can perform measurements of anterior chamber parameters such as the ACD, anterior chamber angle, and anterior chamber volume [88,89]. More recently, the anterior segment optical coherence tomography (AS-OCT) was introduced, which also enables a non-contact assessment of anterior chamber morphometrics [90]. Due to its longer wavelength than is used in Scheimpflug photography, it is less affected by opacities, can be used to examine deeper structures, and is better suited for the analysis of the iridocorneal angle [90,91]. AS-OCT has also been demonstrated to be suitable for the assessment of pIOL positioning [56,92,93,94].

#### 3.4.1. Distance from the Phakic Intraocular Lens to the Endothelium

To prevent endothelial damage, arbitrary safety distances of 2.0 mm from the center of the pIOL to the endothelium and of 1.5 mm from the edge of the pIOL to the endothelium have been proposed [56,90,95]. Using Visante (Carl Zeiss Meditec Inc., Jena, Germany) AS-OCT, Doors et al. examined the distances from the center and the edges of the iris-fixated Artisan and Artiflex pIOLs to the corneal endothelium in 242 eyes and analyzed the relationship between the distances measured and the ECD loss observed with the mean follow-up of 34.12 ± 24.72 months after the pIOL implantation [56]. The authors observed a negative correlation between the endothelial cell loss and the distance from the edge of the pIOL to the endothelium up to 5 years post operation [56]. In a linear mixed-model analysis, the mean observed distance of 1.37 mm resulted in a yearly ECD decrease of 0.98% [56]. A smaller distance from the edge of the pIOL to the endothelium of 1.15 mm almost doubled the yearly rate of endothelial cell loss (1.8%), while a larger distance resulted in a very low predicted yearly decrease in ECD of 0.15% [56]. The authors concluded that the evaluation of anterior chamber morphometrics should be routinely performed alongside ECD measurements to evaluate the safety of pIOLs [56]. They later published a mathematical model to predict ECD changes in iris-fixated pIOL patients in relation to the preoperative ECD, patient age, and the edge distance, which can be used to predict when a certain ECD value will be reached [69]. For example, their model showed that it would take 18 years for a patient with a preoperative ECD of 2000 cells/mm^2^ and an edge distance of 1.43 mm to reach the ECD of 1500 cells/mm^2^ [69].

Although the model by Doors et al. to predict ECD decrease in pIOL patients based on the distance from the pIOL to the endothelium was published more than a decade ago, the practice of measuring of this distance did not gain widespread acceptance in real-world clinical settings. One of the reasons for this could be the equipment and time needed for such measurement, as it is performed manually [69]. Probably for the same reasons, data regarding the distance from the pIOL edge to the endothelium are not available in most long-term clinical studies analyzing endothelial cell loss in patients with pIOLs.

#### 3.4.2. Anterior Chamber Depth

**Figure 5:**
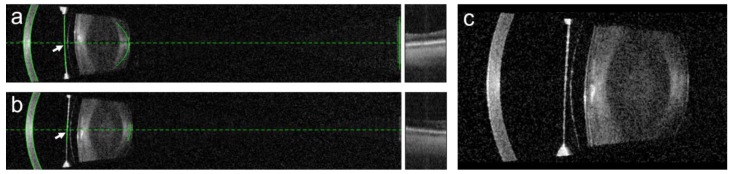
In patients with pIOLs, errors in the anterior chamber depth (ACD) measurement can occur. In the presented case, the ACD was incorrectly measured in both the right (**a**) and the left (**b**) eye of the patient, as the highly reflective surface of the posterior chamber pIOL (**c**) was wrongly identified (arrows) as the anterior surface of the crystalline lens by the automated algorithm of the IOL Master 700. To avoid such errors, it is necessary to visually inspect the biometry measurements in patients with pIOLs.

The direct measurement of the distance between the pIOL and the corneal endothelium can only be performed postoperatively [10,56]. One approach to overcome this limitation is the simulation of the pIOL position using preoperative AS-OCT [69]. Another approach is to measure the ACD, a metric available preoperatively as well as postoperatively, instead of the distance between the pIOL and endothelium, as a shallower anterior chamber generally means that there will be less space available between the pIOL and the corneal endothelium. Although the exact distance may vary to a certain degree in eyes with identical ACD values due to positioning and the characteristics of the pIOL, as well as the anterior segment configuration, the use of the ACD metric has several advantages. It is an automated measurement, making it less time consuming, and is available on a variety of devices, such as Scheimpflug-based and slit-scanning corneal tomographers, optical biometers, and AS-OCT devices [96]. However, care must be taken regarding the definition of the ACD. In general, it can either include or exclude the corneal thickness; thus, these measurements are referred to as the ACD measured from the epithelium or the endothelium, respectively [9]. It is not recommended that the ACD value measured from the epithelium be used as a parameter in pIOL surgery since it is affected by the variability in inter-individual corneal thickness [9,10,97]. When using devices which can only measure the ACD from the epithelium, it is advisable to measure the central corneal thickness as well and subtract its value in order to obtain the ACD value from the endothelium [9]. When analyzing the ACD in patients with pIOLs, care also needs to be taken regarding its posterior limit, which is the anterior surface of the crystalline lens, to avoid inadvertently measuring from the pIOL surface instead [98]. It is essential to visually examine the scans in order to ensure that the structures were identified correctly by the device’s software [98]. Unfortunately, automated ACD measurement errors are common in patients with pIOLs even when using modern biometers (Figure 5) [98].

Several studies have analyzed endothelial cell loss in patients with anterior chamber iris-fixated pIOLs in relation to the ACD [17,19,20,99]. Eldanasoury et al. investigated ECD changes in 57 patients with Artisan and Artiflex iris-fixated pIOLs over a mean follow-up period of 11.8 ± 2.0 years and found a significant negative correlation between endothelial cell loss and the ACD [17]. Eyes with very shallow ACD measurements (3.20 mm or less, measured from the epithelium) experienced a mean yearly ECD decrease of 4.4%, while those with deep anterior chambers (ACD of 3.50 mm or more, measured from the epithelium) had a yearly endothelial cell loss rate of only 0.4% [17]. Jonker et al. presented the results of a prospective study which involved 289 patients implanted with the Artisan Myopia and Artisan Toric iris-fixated pIOLs and followed-up for 94.9 ± 56.5 months and 50.4 ± 46.8 months, respectively [19]. Based on the preoperative ACD measurements and the recorded preoperative and postoperative ECD values, the authors modelled the rate of endothelial cell loss according to the preoperative ACD [19]. They found a 10-year endothelial cell loss of 12.3% when the preoperative ACD, measured from the epithelium, was 3.64 mm, and a 25.3% rate when the ACD was 2.94 mm [19]. The relationship between the ACD and endothelial cell loss was also found in a study by Shajari et al., with higher rates of loss occurring in eyes with ACD measurements of <3.00 mm, measured from the endothelium [99]. Due to the dependency of the ECD decrease on the ACD, it is an important parameter to consider when evaluating the results of clinical pIOL studies [10]. A summary of some of the long-term studies analyzing ECD changes in patients with pIOLs, sorted in ascending order according to the ACD values in these studies, is presented in Table 2.

Until recently, it was not uncommon to implant iris-fixated pIOLs in patients with relatively shallow anterior chambers. For example, the average preoperative ACD, measured from the corneal epithelium, was 3.27 ± 0.31 mm across 293 eyes implanted with Artiflex Myopia pIOLs between 2004 and 2016 in an academic hospital [20]. The emerging evidence of the association between ACD and endothelial cell loss in pIOL patients was reflected in the updated patient selection criteria, defined by the manufacturer of the iris-fixated lenses, currently requiring a minimum ACD of 3.00 mm, measured from the endothelium, in patients undergoing pIOL implantation [100].

The relationship between the ACD and endothelial cell loss in posterior chamber pIOLs has not yet been clearly established. Niu et al. examined the outcomes of 51 eyes with shallow ACD measurements of <2.8 mm, measured from the endothelium, which were implanted with ICLs [102]. Although they reported a relatively high endothelial cell loss of 8.38 ± 0.06% at 15.35 ± 4.90 months postoperatively, they did not find a correlation between the preoperative ACD and endothelial cell loss, and no eyes experienced loss rates of ≥30% [102]. Because of the short follow-up duration in the study, it was not possible to differentiate a chronic endothelial cell loss caused by the pIOL from the acute loss caused by the surgery itself [102]. Yang et al. presented the 4-year outcomes of patients with the model V4c ICL and found no correlation between ECD change and the ACD, but a statistically significant positive correlation between ECD change and the change in anterior chamber angle (i.e., reduction due to the pIOL implantation), the change (i.e., reduction) in anterior chamber volume, and the vault, as well as a negative correlation between the ECD change and the distance between the endothelium and the central ICL [103]. Qian et al. also found a statistically significant correlation between ECD change and the vault, as well as the distance between the endothelium and the central ICL, while the vault was identified as primarily responsible for endothelial cell loss [104]. The prediction and measurement of the vault are discussed in detail in later sections of this review.

When discussing the association between endothelial cell loss and anterior chamber morphometrics, it should be noted that these measurements change with age. Due to increasing crystalline lens thickness, the ACD decreases with age, which is also reflected in the continuously decreasing distance between the pIOL and the endothelium [56,69,82,105]. Long-term data on patients with iris-fixated pIOLs showed a decrease in ACD from preoperatively observed distances of 3.25 ± 0.26 mm to 2.81 ± 0.24 mm 15 years after the pIOL implantation [82]. Therefore, even though most of the eyes in the study had an ACD within the range which is currently considered safe at the time of pIOL implantation, the opposite was the case 15 years later. While most of existing studies have demonstrated relatively low rates of loss in patients with preoperative ACD measurements of ≥3.00 mm, there is a lack of studies with an observation period exceeding 15 years [10]. Because of the effect of the ACD on endothelial cell loss in patients with iris-fixated pIOLs and the fact that the ACD decreases with age, it seems reasonable to consider patient age when determining a safe preoperative ACD value [10,64]. In a young patient, who is expected to have the pIOL implanted for many years, a preoperative ACD of 3.00 mm may not be sufficient to avoid long-term endothelial damage [10]. In patients with iris-fixated pIOLs, it is advisable to routinely measure the ACD as a part of regular follow-up examinations and closely monitor ECD in patients with shallow anterior chambers. Patients with anterior chamber pIOLs and shallow ACDs should be informed about the increased risk of endothelial cell loss, and the decision regarding pIOL explantation should be made in collaboration with the patient after a careful evaluation of all clinical findings, including ECD.

## 4. Addressing the Risk of Cataract

Cataract development is another potential complication in patients with pIOLs [32,72,106,107,108,109,110,111,112,113,114]. Even though it can be treated by performing a cataract surgery, this results in the loss of accommodation. pIOL patients undergo the initial procedure to be spectacle independent, and some of them may not be willing to wear spectacles after the cataract surgery. However, the use of multifocal IOLs may be limited in patients with significant ocular comorbidities and due to the lower accuracy of IOL power calculations in highly myopic and highly hyperopic eyes [115,116,117,118]. Apart from the loss of accommodation, cataract surgery performed on young myopic patients carries a significant risk of retinal detachment [119,120]. Colin et al. reported an incidence of 8.1% over a 7-year follow-up period in highly myopic patients who underwent clear lens exchange [121]. After analyzing data on 1.8 million patients who underwent cataract surgery, Daien et al. found that myopic patients of 40 to 54 years of age had a hazard ratio of 25.02 with regard to the likelihood of them developing a retinal detachment in the first 4 years following cataract surgery [120]. Among all of the examined risk factors, the most important one was high myopia, followed by young age [120]. As retinal detachment can lead to irreversible vision loss in some cases, the risk of retinal detachment after cataract surgery has to be taken into account when considering the risk of cataract development in patients with pIOLs [122].

**Figure 6:**
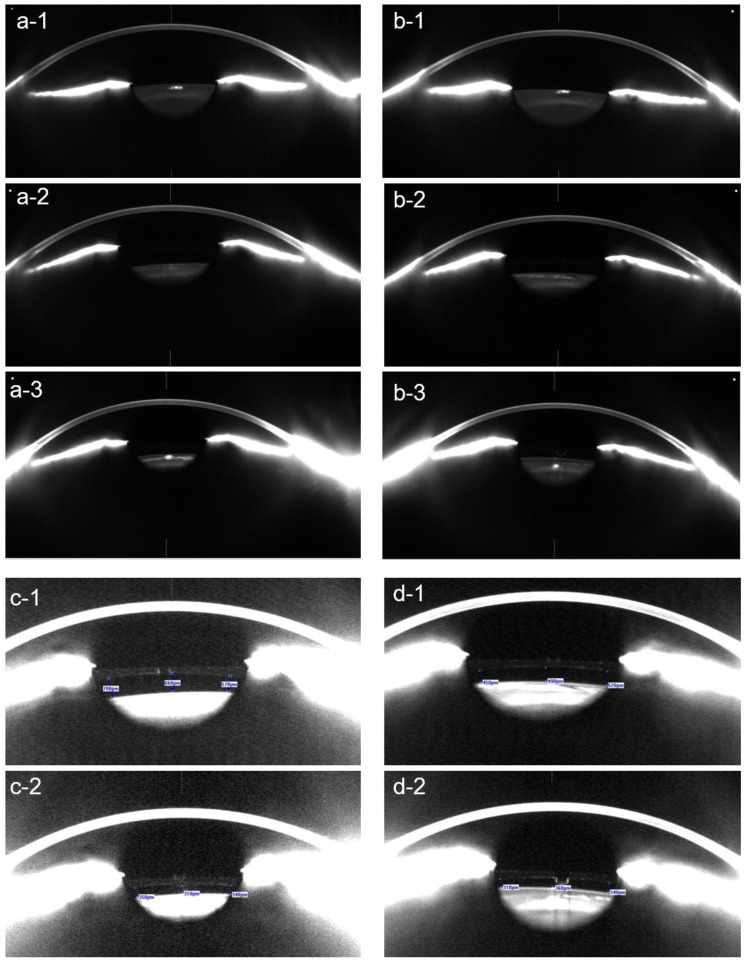
Scheimpflug tomography of the right eye (**a-1**–**a-3**) and the left eye (**b-1**–**b-3**) of a 60-year-old patient before surgery (**a-1**,**b-1**), as well as 3 months (**a-2**,**b-2**) and 4 years (**c-1**,**c-2**) after the implantation of the posterior chamber pIOL. In the right eye, an anterior subcapsular opacity developed 4 years after the pIOL implantation (**a-3**). In the left eye, a slight anterior subcapsular opacity, caused by surgical trauma, was observed within 3 months of the surgery (**b-2**) but did not progress thereafter. In addition to these anterior subcapsular opacities, this patient developed age-related corticonuclear cataracts in both eyes, requiring the combined pIOL explantation and cataract surgery. The vault measurements of the right eye (**c-1**,**c-2**) and the left eye (**d-1**,**d-2**) using Scheimpflug tomography after contrast enhancement revealed a decrease in the vault at 4 years (**c-2**,**d-2**) in comparison to 3 months (**c-1**,**d-1**) following the pIOL implantation.

In contrast to endothelial cell loss, the higher risk of developing cataract is primarily observed in patients with posterior chamber pIOLs, while the anterior chamber pIOLs do not seem to significantly increase the risk [32,72,106,107,108,109,110,111,112,113,114]. In the U.S. Food and Drug Administration clinical trial, which analyzed the outcomes of patients implanted with the V3 and V4 ICL models, it was noticed that some anterior subcapsular lens opacities appeared within the first 90 days following the ICL implantation, while others appeared later, ≥1 year after the surgery [110]. This showed that the pIOL implantation itself can cause a traumatic cataract (Figure 6). Igarashi et al. reported a 6.8% rate of an anterior subcapsular cataract developing immediately after the surgery [107]. Fortunately, these did not progress thereafter and remained asymptomatic [107]. The late-onset cataracts are more concerning, as they are likely caused by the pIOL itself and are more likely to become symptomatic [110]. Most of the reports on cataract development are on patients implanted with the earlier ICL models such as the V4 ICL, which did not have the central port. In these patients, Lackner et al. reported an overall cataract rate of 33.3% and a clinically significant cataract rate of 17.3% after a mean follow-up of 21.9 months [123]. Guber et al. found that lens opacity developed in 40.9% of eyes 5 years after the V4 ICL implantation, and in 54.8% of eyes at 10 years post operation [72]. In patients with ICLs without the central port, lens opacities were shown to increase with time, requiring cataract surgery in 2–4% of eyes within 2 years of ICL implantation, in 2–5% of eyes within 5 years, and in 17–18% of eyes within 10 years [72,109,124,125,126]. The proposed causes for these late-appearing lens opacities include changes in aqueous humor circulation, due to which the flow between the ICL and the crystalline lens is impeded; insufficient nutrition of the lens; intermittent lens touching; and chronic low-grade inflammation [108,127]. In order to enable better aqueous humor circulation, the manufacturer updated the design of the ICL, starting with the model V4c, by including a central hole [112,127]. The initial long-term results suggest a low rate of cataract formation in patients implanted with the V4c ICL model [111,112,128]. After reviewing studies with at least 5 years of follow-up, Wannapanich et al. found the incidence of the anterior subcapsular cataract to be only 0.5%, while that of the nuclear cataract was 0.1% [128].

### The Influence of the Vault on Cataract Development

The decreasing cataract rates in patients with newer ICL models may be attributed not only to the presence of the central port, but also to the increased distance between the ICL and the crystalline lens, also known as the vault [110]. Using UBM, a technique we discussed previously in this review, Jiménez-Alfaro reported that the early V2 ICL model touched the crystalline lens in 72.2% of eyes, with central contact occurring in 16.2% of eyes at some point throughout the 12-month follow-up [129]. Sanders et al. demonstrated that further lens design changes, which increased vaulting away from the crystalline lens, reduced the incidence of lens opacities [109,110]. Nevertheless, some eyes had a low vault even with the newer ICL design [32]. Gonvers et al. demonstrated that cataract development was associated with a low vault in the V3 and V4 ICL models, as all 20 cataracts observed in the study occurred in eyes with central vaulting equal to or less than 0.09 mm [32]. However, the authors recommended aiming for a higher central vault of at least 0.15 mm, as this was found to be sufficient to avoid the peripheral contact between the pIOL and the crystalline lens [32]. Gimbel et al. analyzed the outcomes of 1653 eyes implanted with the V4 ICL model and found anterior subcapsular formation to be negatively correlated with the ACD and positively correlated with age, which can be explained by the lower vaults found in older patients with shallower anterior chambers [106,130]. Currently, an ACD of at least 2.8 mm in myopic patients and of ≥3.0 mm in hyperopic patients, measured from the endothelium, is required in patients undergoing pIOL implantation in Germany [9].

Posterior chamber pIOL design changes reduced but did not eliminate the risk of developing lens opacities. In the newer ICL models with the central hole design, it appears that a low vault is less likely to cause cataracts. Gonzalez-Lopez et al. examined 24 eyes with low central vaults ranging from 9 µm to 94 µm (mean vault ± SD of 52 ± 19 µm) and found an anterior subcapsular cataract in only one eye (4.2%), after a mean ± SD follow-up of 5.8 ± 0.9 years [92]. In this eye, the vault under photopic conditions was 37 µm [92]. However, a study which examined 163 eyes implanted with the V4c ICL and followed-up for at least 24 months found that anterior subcapsular opacity developed in five eyes, and all of them had vault values below 250 µm [104]. Their findings indicate that the vault remains an important factor in anterior subcapsular cataract development, including in patients with the new ICL designs [104].

In order to reduce the risk of cataract, low vaults should be avoided, and the approaches to the ICL size selection and vault prediction are discussed later in this article. In addition, the vault should be monitored postoperatively, as it was found to decrease over time [126,131]. Schmidinger et al. reported a yearly central vault reduction of 20 to 28 µm, with a continuing decrease over the whole follow-up up period of 10 years [126]. In a study of an older ICL design (V4), which did not feature the central hole, it was suggested to explant the ICL immediately if the vault decreases below 150 µm, to prevent cataract formation [106]. In another study, a higher minimum value of 230 µm was recommended, but the authors noted that the insufficient safety distances are only a relative indication for the explantation of a posterior chamber pIOL, as the anterior subcapsular opacities usually develop outside the visual axis and progress slowly [126]. However, the authors stated that a closer follow-up of patients with low vault values is necessary [126]. In his review, Packer summarized that the recommended lower limits of safe vaults found in previous studies were between 50 µm and 250 µm [31]. Low vaults appear to be more tolerable in patients with the newer ICLs with the central port, but a close follow-up of low-vault cases remains necessary as cataract is still more likely to develop in patients with lower vaults [104].

## 5. Addressing the Risk of Ocular Hypertension and Glaucoma

Elevated intraocular pressure is occasionally observed in patients with pIOLs [72,132,133,134,135]. In posterior chamber pIOL patients, intraocular pressure may rise in the first few hours after the ICL implantation because of the narrowing of the anterior chamber angle due to mydriasis [136]. During the early postoperative period, the most common cause for elevated intraocular pressure has been found to be steroid response, followed by retained viscoelastic agent and pupillary block [132,134]. In the older-model ICLs without the central port, pupillary block with anterior chamber angle closure was found to occur due to peripheral iridotomies of insufficient size, which were blocked with pigment debris; due to an oversized ICL; and due to the occlusion of peripheral iridotomies by ICL haptic after the rotation of the lens [133,134]. The newer ICL models feature the central port and therefore do not require peripheral iridotomies [137]. In these ICLs, pupillary block can occur due to the occlusion of the central port by inflammatory debris and viscoelastic agent remaining in the eye [134].

In rare cases, chronically elevated intraocular pressure can occur due to pigment dispersion caused by rubbing between the ICL and the iris [135]. Ye et al. reported a case of a 50-year-old patient who presented for a routine follow-up 8 years after ICMV4 ICL implantation and was diagnosed with advanced pigment dispersion glaucoma, with an intraocular pressure of 41 mmHg [135]. The UBM examination showed direct contact between the ICL and the iris [135].

More commonly, chronically elevated intraocular pressure can occur due to the narrowing of the anterior chamber angle due to a higher vault [104]. Usually, the upper limit for a safe vault is considered to be 1000 µm or 750 µm, but this might not apply to eyes with shallow anterior chambers [31,104]. Qian et al. examined the outcomes of eyes with shallow anterior chambers (ACD < 2.8 mm) and found the mean vault in the eyes with elevated intraocular pressure to be 503 µm, which was higher than the average vault of 382 µm observed in eyes without elevated intraocular pressure [104]. While a vault of 500 µm is generally considered to be optimal, the safety zone for a vault appears to be narrower in eyes with shallow anterior chambers, based on the findings of this study. In Germany, the implantation of pIOLs is contraindicated in myopic eyes with ACD measurements of < 2.80 mm, measured from the corneal endothelium, and in hyperopic eyes with ACD measurements of < 3.00 mm [9]. However, even in eyes with normal ACD and normal average vault, ocular hypertension requiring topical medication was found in 12.9% of eyes implanted with the V4 ICL without the central hole at 10 years postoperatively [72]. Although pigmentation was observed in the iridocorneal angle, it is not specified if other signs of pigment dispersion were present [72].

The findings of the published studies suggest that elevated intraocular pressure is a relatively rare complication in patients with posterior chamber pIOLs, but its rate may increase over a longer follow-up period. Although the reason for this is not yet clearly established, decreases in the anterior chamber angle due to increases in crystalline lens thickness over time might play an important role [104]. In addition, most of the long-term data are on the older ICL models without the central hole, and more studies with a follow-up of at least 10 years are required for the newer ICL models in order to determine the risk of late-onset ocular hypertension and glaucoma.

Much like with the posterior chamber pIOLs, elevated intraocular pressure can occasionally occur due to an ophthalmic viscosurgical device remaining in the eye, steroid response, and pupillary block in patients with anterior chamber lenses [5,138]. To prevent pupillary block, peripheral iridotomies should be performed, as with the older ICL models without the central hole [5]. In addition, chronic inflammation and anterior synechiae can also cause secondary glaucoma [5,138,139].

**Figure 7:**
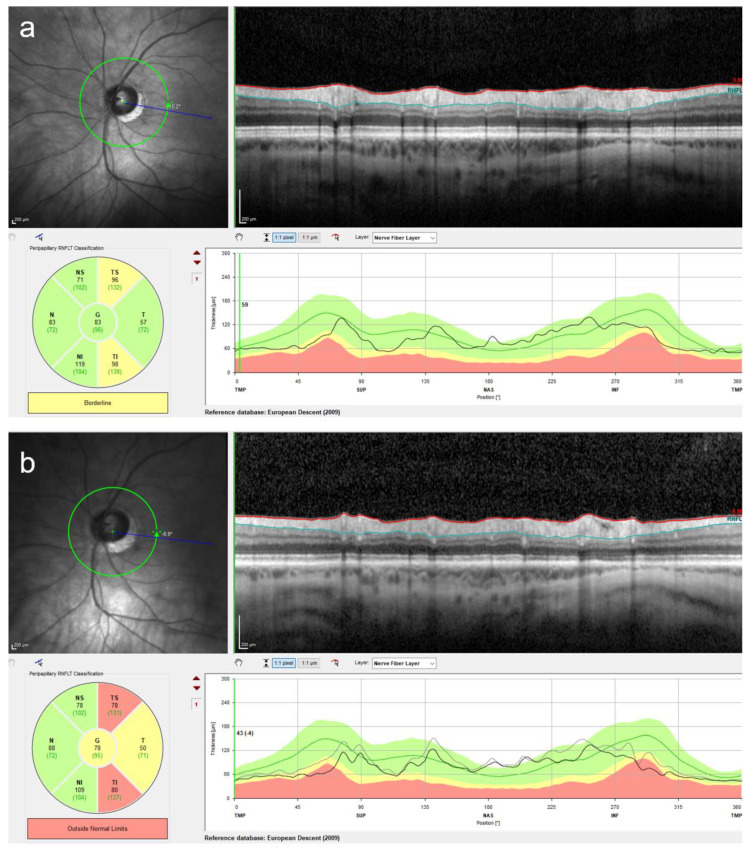
Retinal nerve fiber layer (RNFL) measurement using optical coherence tomography is a useful technique to detect glaucomatous changes. Although the intraocular pressure (IOP) measurement is essential at every follow-up visit in pIOL patients, an increased IOP might be missed due to fluctuations in the IOP. The use of the RNFL measurement can increase the probability of detecting early glaucomatous changes. In this patient, a marked RNFL thinning was observed at four years following the posterior chamber pIOL implantation (**b**) in comparison to the preoperative measurement (**a**). The RNFL measurement was performed using the Spectralis OCT (Heidelberg Engineering, Heidelberg, Germany).

As for other pIOL complications, regular postoperative follow-up examinations are necessary, as a timely diagnosis and treatment is crucial to prevent irreversible vision loss. Intraocular pressure must be measured at each follow-up visit. The slit lamp examination should focus on signs of a narrow anterior chamber angle, pigment dispersion, chronic inflammation, synechiae, and the evaluation of the optic disc. Anterior segment imaging techniques can be used to evaluate the vault and anterior chamber morphometrics in order to assess the risk of developing ocular hypertension in patients with posterior chamber IOLs. Finally, OCT of the retinal nerve fiber layer and visual field examination can be helpful when detecting glaucomatous damage (Figure 7) [135,140].

## 6. Measurement of the Vault

Methods of measuring the central vault include AS-OCT, Scheimpflug photography, and UBM, and these techniques were discussed above [114,130,141,142]. However, it is important to recognize, that the AS-OCT measures significantly higher vault than the UBM, while Scheimpflug tomography shows the lowest values [141]. Almorín-Fernández-Vigo et al. found excellent reproducibility of the vault values measured with both AS-OCT and Scheimpflug tomography, but the AS-OCT values (RTVue 100, Optovue, Freemont, CA, USA) were 128.1 ± 64.6 μm higher than those measured by Scheimpflug tomography (Pentacam) [143]. Therefore, these devices should not be used interchangeably. Most of the analyzed studies used AS-OCT to measure the vault, and care must be taken when applying AS-OCT-based study recommendations on vault values measured using the Scheimpflug tomography. In addition to the objective techniques mentioned, the central vault can be assessed on a slit lamp [144]. However, such a subjective assessment requires experience to be reliable and is not well-suited for research purposes, and therefore, objective methods should be used whenever possible [144]. Although not routinely performed, peripheral vaulting can only be assessed using the UBM, as the optical techniques cannot show the structures behind the iris pigment epithelium [145].

When measuring the vault, attention needs to be paid to the lighting conditions. The vault is not a static parameter and changes according to the pupil size [146,147]. In photopic conditions, Lee et al. found the vault to decrease, on average, by 88.2 µm in the V4 ICL, while the decrease was even more pronounced in the newer ICL models with the central port (147.5 µm) [146]. The authors concluded that the measurements under both mesopic and photopic conditions should be considered when interpreting the postoperative vaulting [146].

The vault can also be measured intraoperatively, using optical coherence tomography (OCT) integrated into the surgical microscope [148]. Although the intraoperative values differ statistically significantly from the postoperative ones, the intraoperative OCT was found to be effective in minimizing postoperative vault surprises [148]. For example, this technology enables immediate management of a high vault in non-toric ICLs, as it can be reduced by rotating the ICL from a horizontal to an oblique or vertical position without the need for a second surgery [148,149,150].

## 7. ICL Vault Prediction

As suboptimal vaults are associated with an increased risk of complications, and the vaulting depends on the size of the ICL, selecting the correct size is necessary to ensure the safety of patients with these lenses [31,137]. The ICL comes in four sizes, and the ICL size is traditionally selected using the manufacturer’s nomogram, based on the ACD and white-to-white distance values [151]. However, suboptimal vaults are not rare when using this approach [137,152]. A recent study of 1745 eyes, implanted with V4c and V5 ICLs, found that while the average vault was 508.5 µm, 8% of the eyes had a low vault (<250 µm), while 9% had a vault of >750 µm and 1% of >1000 µm [152]. The SD of the mean vault was 188 µm [152]. A meta-analysis of 2263 eyes from 24 studies which used the ACD and white-to-white parameters to select the ICL size found comparable vaults, with the mean values ranging from 322 to 594 µm, and the SD from 141 to 268 µm [31]. Assuming a normal distribution of the vault, 16% of eyes would have a vault of 250 µm or less, and 0.4% would have a vault above 1000 µm [31].

Using various preoperative parameters obtained via AS-OCT and Scheimpflug tomography, one study found the horizontal anterior chamber angle-to-angle distance and the crystalline lens rise (the distance between the anterior pole of the lens and the horizontal line connecting the iridocorneal angles), along with the ICL size, its spherical equivalent, and the patient age to be predictors of the vault size. Still, they could only explain 34% of vault variability [153]. Another study identified a high crystalline lens rise and a low ICL spherical equivalent as risk factors for a low vault, while a larger difference between the ICL size and the horizontal anterior chamber angle-to-angle distance resulted in a high vault [154].

One of the reasons for suboptimal vaults achieved in some eyes using the traditional method appears to be a poor correlation between the measured white-to-white distance and the sulcus-to-sulcus diameter, which better represents the actual position of the implanted ICL [155,156,157]. Chen et al. reported the difference between the white-to-white and sulcus-to-sulcus diameters to be larger in eyes with ACD measurements and the white-to-white distances outside a certain range, which could be one of the reasons why suboptimal vaults are achieved in some eyes using the traditional ICL size selection method [158]. In addition, the configuration of the ciliary body can also influence the vault, and a recent study found that the inner diameter of the ciliary body correlates even better with the vault than the sulcus-to-sulcus distance [156,159].

The sulcus-to-sulcus diameter and other ciliary body parameters are used in some of the alternative ICL size selection methods [31,155,156,160]. However, Packer presented a meta-analysis comparing the vault achieved using the traditional methodology and the alternative methods and summarized that both approaches showed similar results in achieving a satisfactory vault [31]. One of the disadvantages of using these parameters for ICL size selection is that they can only be measured using ultrasound-based techniques, which require contact. In addition, the inter-examiner reproducibility of some of the UBM parameters was found to be low [31]. This can be overcome by the use of a very-high-frequency digital ultrasound robotic scanner [156]. Utilizing this technique, Reinstein developed a formula which could achieve the vault within ±300 μm of a target in 94–100% of eyes, with the vault ranging from 250 µm to 1000 µm in 99–100% of eyes [156].

There is growing interest in developing a more accurate vault prediction method using AS-OCT measurements as these devices seem to be becoming increasingly common in ophthalmology clinics. Even though it cannot obtain ciliary body parameters, the AS-OCT is appealing because it allows a fast, repeatable, and non-contact measurement [46,90]. The current AS-OCT-based ICL size calculation formulas appear to achieve similar or slightly better results than the traditional method [152,161,162,163,164]. A direct comparison is difficult because the results of most of the current formulas are presented as a prediction error, while those of the manufacturer’s nomogram are presented as the mean vault achieved. For optimal results when using the AS-OCT-based ICL-sizing formulas, the patient characteristics should ideally be similar to those in the formula-development studies [165]. Most of the new formulas were derived from data from the highly myopic eyes of Asian patients, and the differences in ocular anatomy could limit the accuracy of these formulas in patients of other ethnicities and in lower myopia [165]. To address this issue, Rocamora et al. developed a set of Least Absolute Shrinkage and Selection Operator (LASSO) formulas, based on data from Caucasian eyes with varying degrees of myopia and found these to be more accurate in their patient population when compared to Nakamura 1 and 2 formulas [165]. A summary of some the studies which evaluated alternative ICL calculation methods is presented in Table 3.

Regardless of the accuracy of the method used, the limited choice of ICL sizes remains an issue which prevents achieving more consistent outcomes in terms of the vault [163]. Nakamura et al. noted that in cases where the optimal ICL size is in the boundary area between the two available ICL sizes, the selection of the smaller ICL would result in a vault of <0.2 mm, while the larger ICL would lead to a vault of 0.8 mm [163].

## 8. Conclusions

Design modifications of pIOLs have significantly reduced but have not eliminated the risk of complications. The safety of patients with pIOLs can be further improved by utilizing preoperative and postoperative diagnostic techniques, such as specular microscopy, AS-OCT, Scheimpflug tomography, and UBM. Careful patient selection and regular postoperative follow-up are necessary in order to reduce the complication risk and to enable early intervention in case a complication occurs.

## Figures and Tables

**Table 1:** Long-term endothelial cell density changes in patients implanted with anterior chamber iris-fixated phakic intraocular lenses and with implantable Collamer lenses [10]. Published studies reporting mean endothelial cell density changes throughout at least 5 years of follow-up were included.

Study	Year	pIOL Model	Number of Eyes	Mean ± SD Preoperative ECD (cells/mm^2^)	ECD at 1 Year Postoperatively (cells/mm^2^)	ECD at 5 Years Postoperatively (cells/mm^2^)	ECD at 10 Years Postoperatively (cells/mm^2^)
**Anterior chamber iris-fixated phakic intraocular lenses**							
Benedetti et al. [16]	2007	Artisan	49	2616 (median value)	2523 (median value)	2379 (median value)	-
Bouheraoua et al. [59]	2015	Artisan	68	2629 ± 366	2464 ± 334	2250 ± 454	-
Castro de Luna et al. [14]	2019	Artiflex	53	3107 ± 428	3028 ± 436	2867 ± 460	2673 ± 453 (*n* = 18)
Eldanasoury et al. [17]	2019	Artisan, Artiflex	90	2645 ± 200	-	-	Approx. 12 years: 1751 ± 730
Güell et al. [18]	2008	Artisan	Group 1: Artisan Myopia Model 204, *n* = 101. Group 2: Artisan Myopia Model 206, *n* = 173. Group 3: Artisan Hyperopia Model 203, *n* = 41. Group 4: Artisan Toric, *n* = 84.	Group 1: 2836 ± 398 Group 2: 2755 ± 362 Group 3: 2735 ± 355 Group 4: 2632 ± 543	Group 1: 2598 ± 350 Group 2: 2643 ± 414 Group 3: 2600 ± 442 Group 4: 2673 ± 439	Group 1: 2514 ± 529 Group 2: 2454 ± 588 No data from groups 3 and 4	-
Jonker et al. [20]	2018	Artiflex	Artiflex Myopia: *n* = 293 Artiflex Toric: *n* = 188	Artiflex Myopia: 2739 ± 286 Artiflex Toric: 2769 ± 370	Artiflex Myopia: 2657 ± 352 (*n* = 202) Artiflex Toric: 2669 ± 426 (*n* = 131)	Artiflex Myopia: 2480 ± 369 (*n* = 137) Artiflex Toric: 2488 ± 360 (*n* = 63)	-
Jonker et al. [19]	2018	Artisan	507	Artisan Myopia: 2670 ± 365, *n* = 381 Artisan Toric: 2695 ± 359, *n* = 126	-	Artisan Myopia: 2588 ± 425, *n* = 193 Artisan Toric: 2270 ± 406, *n* = 40	Artisan Myopia: 2302 ± 451, *n* = 127 Artisan Toric: 2009 ± 475, *n* = 20
Kwitko et al. [12]	2021	Artisan, Artiflex	195	Myopia: 2515 ± 371 Hyperopia: 2556 ± 457	-	-	Approx. 7 years: Myopia: 2377 ± 353 Hyperopia: 2436 ± 190
Marta et al. [82]	2022	Artiflex	217	2849 ± 393	-	2693 ± 446	15 years: 2113 ± 446
Morral et al. [79]	2016	Artisan, Artiflex	58 in total (2 study groups of equal size, with both lens models analyzed together)	2836 ± 379 and 2759 ± 365	2827 ± 234 and 2845 ± 438	2723 ± 278 and 2649 ± 398	2654 ± 409 and 2543 ± 419
Nemcova et al. [24]	2021	Verisyse, Veriflex	85	2588 ± 285	2430 ± 312	2175 ± 298	12 years: 2091 ± 312
Papa-Vettorazzi et al. [28]	2022	Artiflex	76	2935 ± 359	2818 ± 350	-	Approx. 11 years: 2620 ± 453
Saxena et al. [64]	2008	Artiflex	318	2817 ± 356	2813 ± 426, *n* = 251	2581 ± 293, *n* = 51	7 years: 2451 ± 256, *n* = 13
Tahzib et al. [80]	2007	Artisan	89	2817 ± 359	2928 ± 351	-	2800 ± 292
Yaşa and Ağca [81]	2018	Verisyse, Veriflex	Verisyse: *n* = 47 Veriflex *n* = 50	Verisyse: 2681 ± 275 Veriflex: 2656 ± 270	Verisyse: 2599 ± 242 Veriflex: 2575 ± 253	Verisyse: 2482 ± 242 Veriflex: 2460 ± 282	-
Yildirim et al. [83]	2021	Artiflex	52	2712 ± 272	2610	2440	6 years: 2411 ± 281
**Implantable Collamer lenses**							
Alfonso et al. [58]	2011	V4	188 (5-year data: *n* = 50)	2695 ± 467	-	2495 ± 357 (*n* = 50)	-
Alfonso et al. [15]	2019	V4c	146	2657 ± 362	2696 ± 358	2645 ± 359	-
Choi et al. [70]	2019	V4	110	2889 ± 239	2893 ± 303	-	2749 ± 300 (*n* = 71)
Guber et al. [72]	2016	V4	133	2300 (median value)	2300 (median value), *n* = 100	2200 (median value), *n* = 106	2393 (median value), *n* = 75
Igarashi et al. [73]	2014	V4	41	2819 ± 295	2756 ± 337	-	8 years: 2626 ± 207
Lee et al. [74]	2016	V4	281	2898 ± 404	2835 ± 337	2726 ± 227	7 years: 2712 ± 369
Moya et al. [61]	2015	V3, V4	144	2587 ± 320, *n* = 85	2434 ± 290, *n* = 73	-	12 years: 2071 ± 362, *n* = 104
Nakamura et al. [75]	2019	V4	114	2740 ± 362	2766 ± 339	2725 ± 298	2581 ± 345
Papa-Vettorazzi et al. [62]	2022	V4b	45	2930 ± 441	2943 ± 475	-	Approx. 11 years: 2731 ± 623
Pesando et al. [77]	2007	V1, V2, V3, V4	59	2696 ± 298	-	-	2437 ± 243

ECD—endothelial cell density; SD—standard deviation.

**Table 2:** Anterior chamber depth and long-term endothelial cell loss: an overview of published studies reporting ≥5-year endothelial cell losses in patients with iris-fixated anterior chamber phakic intraocular lenses, sorted in an ascending order according to the anterior chamber depth [10]. An anterior chamber depth of 3.0 mm or higher, measured from the endothelium, is currently required by the manufacturer of the iris-fixated lenses [100].

Study	Year	pIOL Model	Follow-Up (Years)	Number of Eyes (n)	Mean ± SD Anterior Chamber Depth *	Yearly Chronic Endothelial Cell Loss ^+^	Endothelial Cell Loss at 5 Years Postoperatively ^+^	Endothelial Cell Loss at 10 Years Postoperatively ^+^	Endothelial Cell Loss of 25% or More
Minimum preoperative anterior chamber depth lower than 3.0 mm *									
Jonker et al. [20]	2018	Artiflex	5	Artiflex Myopia: *n* = 293 Artiflex Toric: *n* = 188	Artiflex Myopia: approx. 2.73 mm (3.27 ± 0.31 mm, measured from the epithelium) Artiflex Toric: approx. 2.70 mm (3.24 ± 0.35 mm, measured from the epithelium)	Artiflex Myopia: approx. 2.3% Artiflex Toric: approx. 2.2%	In comparison with 6-month data: Artiflex Myopia: 10.5%, *n* = 137 Artiflex Toric: 10.2%, *n* = 63	n/a	Artiflex Myopia: 5 years—4.4% Artiflex Toric: 5 years—4.3%
Bouheraoua et al. [59]	2015	Artisan	5	68	Approx. 2.90 mm (3.44 ± 0.41 mm, measured from the epithelium)	1.7%	15.2%	n/a	n/a
Eldanasoury et al. [17]	2019	Artisan, Artiflex	9–17	90	2.91 ± 0.33 mm	2.3%	n/a	Mean follow-up of 12 years: 26.7 ± 27.6%	n/a (an explantation rate of 26% due to endothelial cell loss)
Jonker et al. [19]	2018	Artisan	10	507	Artisan Myopia: approx. 3.14 mm (3.68 ± 0.34 mm, measured from the epithelium) Artisan Toric: approx. 2.95 mm (3.49 ± 0.35 mm, measured from the epithelium)	Artisan Myopia: approx. 1.8% Artisan Toric: approx. 2.3%	Artisan Myopia: 4.1%, *n* = 193 Artisan Toric: 11.9%, *n* = 40	Artisan Myopia: 11.5%, *n* = 127 Artisan Toric: 18.5%, *n* = 20	Artisan Myopia: 5 years—1.8% 10 years—7.9% Artisan Toric: 5 years—3.2% 10 years—6.3%
Saxena et al. [64]	2008	Artiflex	≤7	318	Approx. 3.16 mm (3.70 ± 0.30 mm, measured from the epithelium)	n/a	8.3%, *n* = 5	n/a	n/a
Papa-Vettorazzi et al. [28]	2022	Artiflex	≥10	76	3.21 ± 0.26 mm	1.0%	n/a	In comparison with 1-year data: 8.9 ± 11.9%	n/a
Marta et al. [82]	2022	Artiflex	≤15	217	3.25 ± 0.26 mm	1.0–1.7%	Artiflex Myopia: 7.1 ± 15.6% Artiflex Toric: 1.3 ± 13.8%	Artiflex Myopia: 17.3 ± 16.4% Artiflex Toric: 16.5 ± 12.8%	5 years—7.2% 10 years—24.3% 15 years—35.1%
Monteiro et al. [22]	2021	Artiflex	6	177	3.26 ± 0.24 mm	Approx. 1.2%	6-year data, in comparison with 1-year data: 6.0%	n/a	n/a
Royo et al. [63]	2022	Artiflex	8	Artiflex Myopia: *n* = 47 Artiflex Toric: *n* = 20	Artiflex Myopia: 3.35 ± 0.23 mm Artiflex Toric: 3.19 ± 0.21 mm	n/a	Artiflex Myopia: 5.3%, *n* = 36 Artiflex Toric: 6.7%, *n* = 13	n/a	0%
Nemcova et al. [24]	2021	Verisyse, Veriflex	12	85	3.30 ± 0.23 mm	n/a	15.8%	12-year data: 19.1%	5 years—15% 12 years—20%
Güell et al. [18]	2008	Artisan	5	Artisan Myopia: *n* = 274; Artisan Hyperopia *n* = 41; Artisan Toric, *n* = 84.	n/a	n/a	10.9–11.3% Artisan Hyperopia/Toric: n/a	n/a	n/a
Morral et al. [79]	2016	Artisan, Artiflex	10	Group 1 *: *n* = 29 Group 2 *: *n* = 29	n/a	n/a	Group 1: 4.0 ± 6.3% Group 2: 4.0 ± 5.3%	Group 1: 6.4 ± 8.0% Group 2: 7.8 ± 6.8%	n/a
Minimum preoperative anterior chamber depth of 3.0 mm or higher *									
Yaşa and Ağca [81]	2018	Verisyse, Veriflex	5	Verisyse: *n* = 47 Veriflex *n* = 50	Verisyse: 3.27 ± 0.21 mm Veriflex: 3.32 ± 0.26 mm	Verisyse: 1.0–1.2% Veriflex: 1.1–1.2%	Verisyse: 7.4% Veriflex: 7.6%	n/a	0%
Chebli et al. [60]	2018	Artisan	5.4 ± 3.0	113	3.42 ± 0.26 mm	0.9%	n/a	12.1% (*n* = 16)	n/a
Yildirim et al. [83]	2021	Artiflex	6	52	n/a	n/a	6-year data: 11.1%, *n* = 42	n/a	0%
Kwitko et al. [12]	2021	Artisan, Artiflex	1–17	195	n/a	Myopia: 0.8% Hyperopia: no statistically significant loss	Mean follow-up of 7 years: Myopia: 5.5%. Hyperopia: no statistically significant loss.	n/a	n/a

*—measured from endothelium. In cases where the anterior chamber depth was measured from the epithelium, 541 µm was subtracted to correct for the central corneal thickness [101]. The studies which did not specify how the anterior chamber depth was measured were not included. ^+^—negative figures indicate an increase in endothelial cell density. n/a—data not available.

**Table 3:** An overview of studies evaluating alternative ICL sizing methods.

Study	Year	Number of Patients and Eyes	ICL Model	The Actual ICL-Sizing Method Used in the Surgeries	Formula Development Method	Parameters Used in Calculation	Diagnostic Modalities Used to Obtain the Parameters Used in the Final Formula	Main Results
Dougherty et al. [160]	2010	73 eyes of 48 patients (nomogram development)	ICL *	ICL size according to UBM measurements	Multiple regression analysis	STS, ICL power	High-frequency UBM VuMax-II (Sonomed, Inc.)	Mean ±SD vault: 340 ± 174 µm (range 90–952 µm); vault between 100 µm and 700 µm in 93.1% of eyes.
Kojima et al. [155]	2012	Development dataset: 47 eyes of 25 patients in development Validation dataset: 81 eyes of 43 patients	ICL *	Manufacturer’s nomogram in the development stage and the developed formula in the validation stage	Stepwise multiple regression analysis	ACD, STS, STSL	ACD obtained using IOL Master (Carl Zeiss Meditec), STS and STSL obtained using high-frequency UBM VuMax-II	Mean ± SD prediction error: 60 ± 290 µm ^+^; vault between 150 µm and 1000 µm in 88.9% of eyes; vault between 250 µm and 750 µm in 74.1% of eyes; no eyes with a vault of <150 µm and 11.1% with a vault of >1000 µm.
Malyugin et al. [166]	2015	29 eyes of 16 patients (sizing evaluation)	ICL *	Distance from iris pigment end to iris pigment end	ICL size selected according to the distance from iris pigment end to iris pigment end	Iris pigment end to iris pigment end	AS-OCT Visante (Carl Zeiss Meditec)	Mean ±SD vault: 0.53 ± 0.18 mm (range 0.24–0.84 mm); 55.2% had a vault of 0.35 to 0.70 mm; 20.1% had a vault of 0.24 to 0.34 mm.
Oleszko et al. [164]	2020	81 eyes of 43 patients	V4c	Manufacturer’s nomogram	Partial least squares regression algorithm	ATA, ACD, LE, Km, LT, AL, Rm, ACV, ICL size, MRSE	ATA, ACD, lens elevation obtained with AS-OCT (Visante); Km, LT, AL obtained with SS-OCT biometer (IOLMaster 700; Carl Zeiss Meditec); Rm, ACV obtained with Scheimpflug camera (Pentacam AXL; Oculus Optikgeräte)	Mean ±SD prediction error: 2 ± 110 µm. Prediction error: within ±100 μm in 73% of eyes, within ±200 μm in 90% of eyes, within ±300 μm in 100% of eyes.
Nakamura et al. [163]	2020	81 eyes of 41 patients for formula development; 68 eyes of 42 patients for formula validation	V4c	NK formula	Stepwise multiple regression analyses	ACW, CLR	AS-OCT CASIA2 (Tomey)	NK-formula V2: mean ± SD absolute prediction error: 201 ± 146 µm, mean ± SD achieved vault: 670 ± 223 µm (range 235 to 1293 µm), a vault of <250 µm in 1.5% of eyes, a vault of >1000 µm in 7.3% of eyes.
Igarashi et al. [162]	2021	121 eyes of 65 patients	ICL with central port	KS formula	KS formula, based on multiple regression analysis [167]	ATA, ICL size	AS-OCT CASIA2	Mean ±SD prediction error: 2.6 ± 184.9 µm Mean ± SD vault: 423.6 ± 183.3 μm (range 39 to 862 μm)
Kamiya et al. [152]	2021	1745 eyes of 1745 patients	V4c and V5	Manufacturer’s nomogram	Machine learning: 1—support vector regressor; 2—gradient boost regressor; 3—random forest regressor; 4—linear regressor.	Age, sex, sphere, cylinder, MRSE, best-corrected visual acuity, ICL model (non-toric/toric), ICL size, WTW, ACD, ATA, CLR, ACW, LV, central corneal thickness, AOD500, TIA500	AS-OCT CASIA2	Mean prediction error ±95% limits of agreement: 1—14.6 ± 174.4 µm; 2—0.8 ± 137.1 µm; 3—0.9 ± 134.3 µm; 4—0.1 ± 142.2 µm. Mean ± SD absolute prediction error: 1—131.4 µm; 2—103.0 µm; 3—99.6 µm; 4—107.2 µm.
					manufacturer’s nomogram	ACD, WTW		Mean ± SD vault: 508.5 ± 188.0 µm
Reinstein et al. [156]	2022	147 eyes in total: 42 eyes in stage 1; 36 eyes in stage 2; 69 eyes in stage 3.	V4c	Kojima nomogram [155] in stage 1; Reinstein formula v1.0 in stage 2; Reinstein formula v2.0 in stage 3.	Stepwise multivariate regression analysis	Stage 1: STS, STSL, ACD; Stages 2 and 3: ICL size, CBID, STSL, ICL power, SPD.	STS, STSL, CBID measured using very high-frequency digital ultrasound robotic scanner Artemis Insight 100 (ArcScan, Inc., Golden, CO, USA); SPD measured using the Procyon P3000 Dynamic Binocular Pupillometer (Keeler Instruments, Inc., Malvern, PA, USA); WTW, ACD measured using the MS-39 OCT (CSO).	Mean ± SD prediction error: 47 ± 124 µm Achieved vault within ±100, ±200, and ±300 μm of target: Stage 1: 33%, 50%, and 74% of eyes; Stage 2: 58%, 89%, and 100% of eyes; Stage 3: 62%, 84%, and 94% of eyes. Vault <250 or >1000 µm: Stage 1: 12% of eyes; Stage 2: 0% of eyes; Stage 3: 1% of eyes.
Di et al. [161]	2023	300 eyes of 300 patients: 150 eyes for formula establishment and 150 eyes for validation	V4c	Manufacturer’s nomogram	Multiple linear regression analysis	ACD, ATA, ICL size	AS-OCT Visante	Mean ± SD absolute prediction error: 135.1 µm; 20.7% of eyes with mean absolute prediction error greater than 200 µm; root mean square error 157.46 µm; 95% CI −313.2 to 305.9 µm.
Rocamora et al. [165]	2023	115 eyes of 59 patients in the training set and 37 eyes of 19 patients in the test set.	V4c	Selected at the discretion of the surgeon, guided by the manufacturer’s nomogram and the Nakamura 1 and 2 formulas	Least Absolute Shrinkage and Selection Operator (LASSO)	PD, CT, TKm, WTW, LT, ACD, posterior Km at 3 mm zone, anterior Km at 5 mm zone, CV, CLR, SS to SS distance, horizonal visible iris diameter, ICL spherical equivalent, MRSE, manifest refraction cylinder, ICL diameter, age.	PD, posterior Km at 3 mm zone, anterior Km at 5 mm zone, CV, CLR, SS to SS distance, horizonal visible iris diameter obtained using AS-OCT MS-39; CT, TKm, WTW, LT, ACD measured using optical biometer IOL Master 700.	Mean ± SD absolute prediction error: 145.6 ± 100.6 µm for AS-OCT-based model 144.1 ± 107.9 µm for optical biometer-based model 132.0 ± 86.6 µm for the combined model (AS-OCT and optical biometer parameters) Absolute prediction error <500 µm in 97.3–100% of eyes.
Shen et al. [168]	2023	6297 eyes of 3536 patients. Data randomly divided into training set and test set (ratio of 8:2)	V4c	Selected according to the preoperative measurements by a technician	Machine learning: 1—Random Forest, 2—Gradient Boosting, 3—XGBoost.	ICL size, ACD, pupil size, ACA, CT, AL, the time after surgery, K2 value, K2 axis, K1 value, K1 axis, WTW, sphere, sphere of ICL, cylinder, cylinder of ICL, spherical equivalent of ICL, type of ICL	K1, K2, ACD, ACA, pupil size, CT WTW obtained using Pentacam HR (Oculus Optikgeräte); AL measured using IOL Master.	Accuracy (95% CI) of achieving a normal vault, defined as 250–750 µm: 1—0.828 (0.819 to 0.836); 2—0.815 (0.809 to 0.821); 3—0.802 (0.791 to 0.813). RMSE (95% CI): 1—159.026 (155.988 to 162.065); 2—161.862 (158.963 to 164.761); 3—162.527 (159.163 to 165.890).

*—older ICL model without the central port; ^+^—converted to µm for consistency. Abbreviations: anterior chamber volume (ACV), anterior chamber width (ACW), anterior chamber angle (ACA), angle-to-angle distance (ATA), anterior chamber depth (ACD), an average of nasal and temporal angle open distance at 500 μm (AOD500), axial length (AL), ciliary body inner diameter (CBID), crystalline lens rise (CLR), corneal thickness (CT), corneal volume (CV), mean keratometry value (Km), lens elevation (LE), lens thickness (LT), lens vault (LV), manifest refraction spherical equivalent (MRSE), posterior cornea mean curvature (Rm), pupillary diameter (PD), standard deviation (SD), scotopic pupil diameter (SPD), scleral spur (SS) sulcus-to-sulcus diameter (STS), distance between STS plane and anterior crystalline lens surface (STSL), root mean square error (RMSE), an average of nasal and temporal trabecular iris angle at 500 μm (TIA500), mean total keratometry (TKm), ultrasound biomicroscopy (UBM), white-to-white distance (WTW), 95% confidence interval (95% CI).

## Data Availability

All data generated or analyzed during this study are included in this published article.
